# Acute Hemorrhagic Leucoencephalitis (AHLE): A Rare CNS Presentation of Mycoplasma pneumoniae

**DOI:** 10.7759/cureus.30921

**Published:** 2022-10-31

**Authors:** S.K. Jakaria Been Sayeed, Md Moniruzzaman, Reaz Mahmud, Mohammad B Rashid, Subir Chandra Das

**Affiliations:** 1 Medicine and Rheumatology, National Institute of Neurosciences & Hospital, Dhaka, BGD; 2 Stroke Unit, National Institute of Neurosciences & Hospital, Dhaka, BGD; 3 Neurology, Dhaka Medical College Hospital, Dhaka, BGD; 4 Clinical Neurology, National Institute of Neurosciences & Hospital, Dhaka, BGD

**Keywords:** ahle, cns presentation, weston-hurst syndrome, mycoplasma pneumoniae, hemorrhagic leucoencephalitis

## Abstract

Acute hemorrhagic leucoencephalitis (AHLE) is a rare inflammatory disease of the brain. Literature on the presentation and management of this rare disease is limited. A *Mycoplasma pneumoniae* infection is considered a possible trigger for acute hemorrhagic leucoencephalitis (Weston-Hurst syndrome). We report a case of a 58-year-old man presenting with an altered level of consciousness following a history of acute respiratory tract infection. He had also clinical and laboratory features of disseminated intravascular coagulation (DIC). Brain imaging was suggestive of hemorrhagic encephalitis involving both the fronto-temporo-parieto-occipital lobes involving the cortical, subcortical, and splenium of the corpus callosum and the posterior limb of the right internal capsule. Antibodies against Mycoplasma were strongly positive in serum. The patient was treated with fresh frozen plasma, broad-spectrum antibiotics, and methylprednisolone. However, the patient died after 17 days of hospitalization probably due to multiorgan failure and brain herniation.

## Introduction

Acute hemorrhagic leucoencephalitis (AHLE) is a severe, rapidly progressive but rare form of acute disseminated encephalomyelitis (ADEM). The hemorrhagic lesion may involve the white matter of the cerebrum, brain stem, cerebellum, or spinal cord. The histologic hallmark is perivascular polymorphonuclear infiltrates, small vessel necrosis, and demyelination. It is also known as Weston-Hurst syndrome, according to the name of the first descriptor [1]. The etiology of AHLE is still unknown. An autoimmune reaction due to molecular mimicry with some viral or bacterial pathogen like Herpes simplex (HSV), Varicella zoster (VZV), influenza virus, Epstein bar virus, mumps, *Mycoplasma pneumoniae*, coronavirus disease 2019 (COVID-19), and dengue virus may trigger the reaction [1-4]. Genetic polymorphisms like Ran Binding Protein (RANBP)-2 variant may predispose autoimmune responses [5]. In contrast to ADEM, it usually affects adults, especially males. The typical presentation of AHLE is a short history of respiratory tract infection followed by a multifocal neurological deficit according to the site of the lesion, features of raised intracranial pressure, the rapid development of coma, and even as a cause of death [1,6]. *Mycoplasma pneumoniae* is a common pulmonary pathogen that may cause pneumonia with varying severity. The extrapulmonary presentation of the bacteria sometimes overshadows its respiratory manifestations. Although rare, nervous system manifestations, like Guillain-Barré syndrome (GBS), transverse myelitis, encephalitis, optic neuritis, and ADEM, may sometimes be associated with this bacteria. The autoimmune reaction, meaning the development of autoantibody, which reacts with myelin and neural tissue, is responsible for such disease development [6]. There are few case reports of *Mycoplasma pneumoniae* triggering autoimmune hemorrhagic encephalitis in the medical literature, and none from Bangladesh. Here, we report a confirmed *Mycoplasma pneumoniae*, causing necrotizing hemorrhagic encephalopathy involving deep cerebral white matter.

## Case presentation

A 58-year-old diabetic, male, was admitted to our hospital with complaints of fever, cough, and shortness of breath for 10 days followed by an altered level of consciousness for the last 1 day. The fever was high-grade, remittent and the cough was productive. Initially, the patient was admitted to a local hospital where he was given antibiotics for seven days for pneumonia. However, his condition deteriorates further due to increased breathlessness. On his 10th day of illness, he became drowsy, icteric, pale, and unable to follow any command. Therefore, he was referred to our hospital for further evaluation. On examination, the patient was found obtunded (Glasgow Coma Scale (GCS) 9/15), anemic, icteric, hypotensive, hypoxic (94%), oliguric, and still had a fever. Signs of meningeal irritation were absent, the optic fundus was normal, and the plantar response was bilaterally extensor. Systemic examinations revealed bronchial breath sound and crepitation in both lungs field. Stigmata of chronic liver disease were absent. Laboratory investigations revealed reduced hemoglobin, high mean corpuscular volume (MCV), increased reticulocyte count, evidence of hemolysis in peripheral blood film, positive Coombs test, high C-reactive protein (CRP), elevated bilirubin, liver enzyme, and lactate dehydrogenase (LDH). A tracheal aspirate was taken, and a culture was done; however, the culture revealed no growth. Blood culture was found negative but antibodies against Mycoplasma were found strongly positive in the enzyme-linked immunoassay (ELISA) test. The coagulation profile revealed high prothrombin time (PT), activated partial thromboplastin time (APTT), and D-dimer. CSF study was done after correction of the coagulation profile and revealed elevated protein and glucose, absent oligoclonal band, and PCR for herpes simplex virus (HSV), varicella zoster, Cytomegalovirus (CMV), and tuberculosis was found negative (Table 1).

**Table 1:** Laboratory characteristics of the patient during admission at NINS AST- aspartate deaminase, ALT- alanine deaminase, AFB- acid-fast Bacilli, APTT- activated partial thromboplastin time, CRP- C-reactive protein, C/S- culture & sensitivity, CSF- cerebrospinal fluid, DGI- dark ground immunofluorescence, FDP- fibrin degradation product, HAV- hepatitis A virus, HBsAg- hepatitis B virus surface antigen, HCV- hepatitis C virus, HEV- hepatitis E virus, LDH- lactate dehydrogenase, NINS- National Institute of Neurosciences & Hospital

Variable	Value	Reference
Hemoglobin	6.8 g/dl	12.5-16 g/dl
White cell count	19850/mm3	4-11 / mm3
Neutrophil	92 %	40-75 %
Lymphocyte	6 %	20-40 %
Platelet	35000 / mm3	150- 450 /mm3
MCV	102 fL	76-96 fL
Reticulocyte count	4.3 %	0.5-2 %
Peripheral blood film	macrocyte, spherocyte, few microcytes, fragmented RBC, thrombocytopenia	
ESR	140 mm in 1^st^ hour	0-10 mm in 1^st^ hour
CRP	279.9 mg/L	< 5 mg/L
Coombs test	Positive	
LDH	881 U/L	100-250 U/L
ALT	287 U/ L	10-40 U/L
AST	210 U / L	8 -40 U / L
S. Bilirubin	4.8 mg/dl	0.3-1.2 mg/dl
S Creatinine	1.7 mg/dl	0.5- 1.3 mg /dl
HBsAg, Anti HCV, Anti HAV, Anti HEV, Anti dengue IgM & IgG	Negative	
Blood C / S	No growth	
Urine C/S	No growth	
Urine DGI for leptospira	Negative	
Antileptospira antibody (IgM, IgG )	Negative	
Antimycoplasma Antibody (Ig M, IgG)	≥ 1: 640	< 1: 80
RT-PCR for Covid -19	Negative	
CSF Study		
Protein	58 mg/dl	15-45 mg/dl
Glucose	125 mg/dl	40-80 mg/dl
WBC	05	0-5 /mm3
Lymphocyte	05	
Gram, AFB stain	Negative	
PCR for Herpes simplex, Varicella zoster, Cytomegalo virus, and Tuberculosis	Negative	
S. Sodium (Na)	130 mmol/L	135-145 mmol/L
S. Potassium (K)	4.5 mmol/ L	3.5-4.5 mmol/L
Prothrombin time	100 second	12-17 second
APTT	120 second	28-36 second
D-dimer	15.9 µg/ml	< 0.5 µg/ml
FDP	99.3 µg/ml	< 0.5 µg/ml

**Figure 1:**
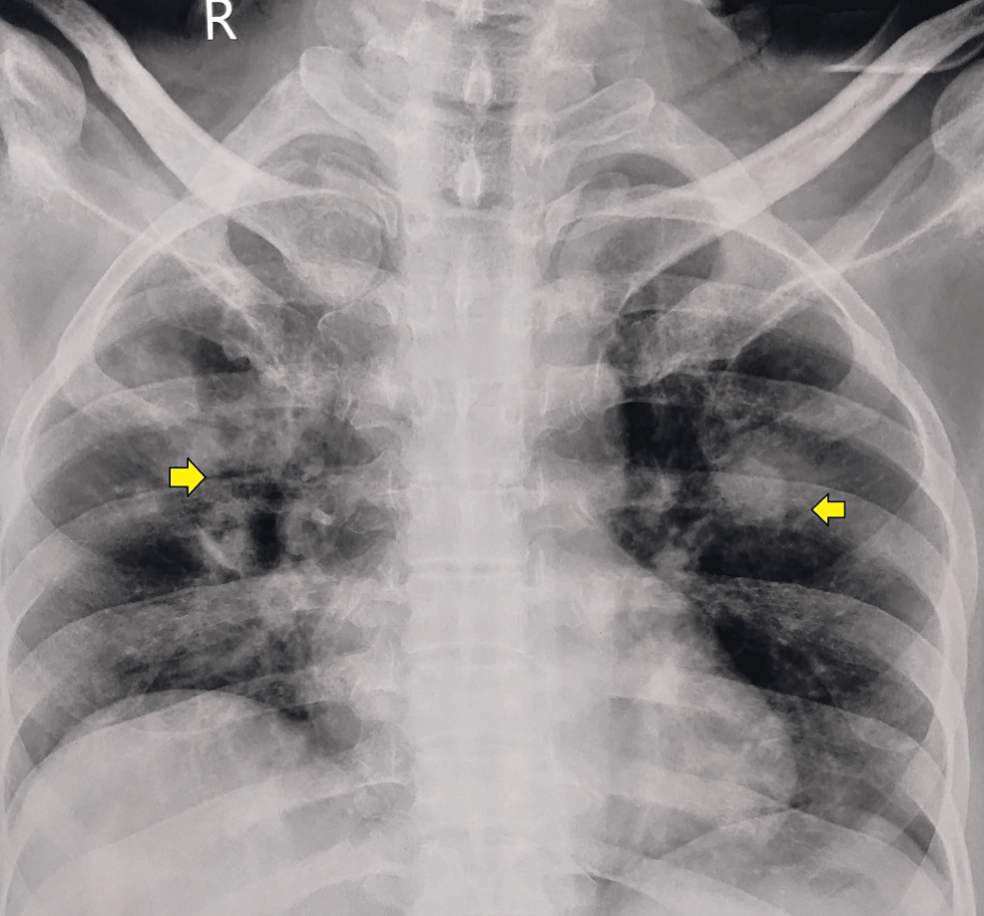
Chest X-ray shows consolidation in both lungs (yellow arrows)

Chest X-ray revealed bilateral consolidation (Figure 1).

**Figure 2:**
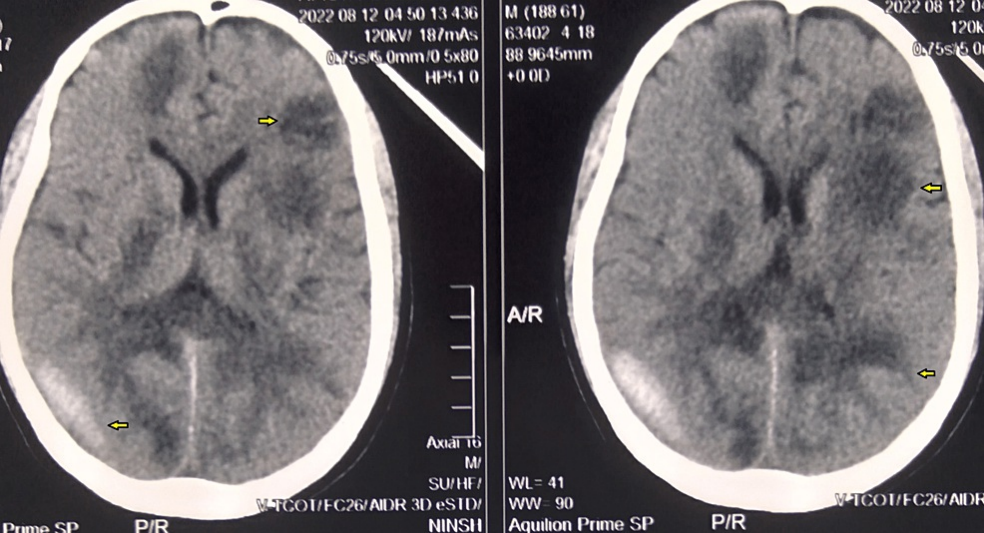
CT scan head (non-contrast} showing multiple mixed-density lesions (yellow arrows) all over the cerebral cortex

CT scan head revealed intracerebral hemorrhage with multiple mixed-density lesions with perilesional edema (Figure 2).

**Figure 3:**
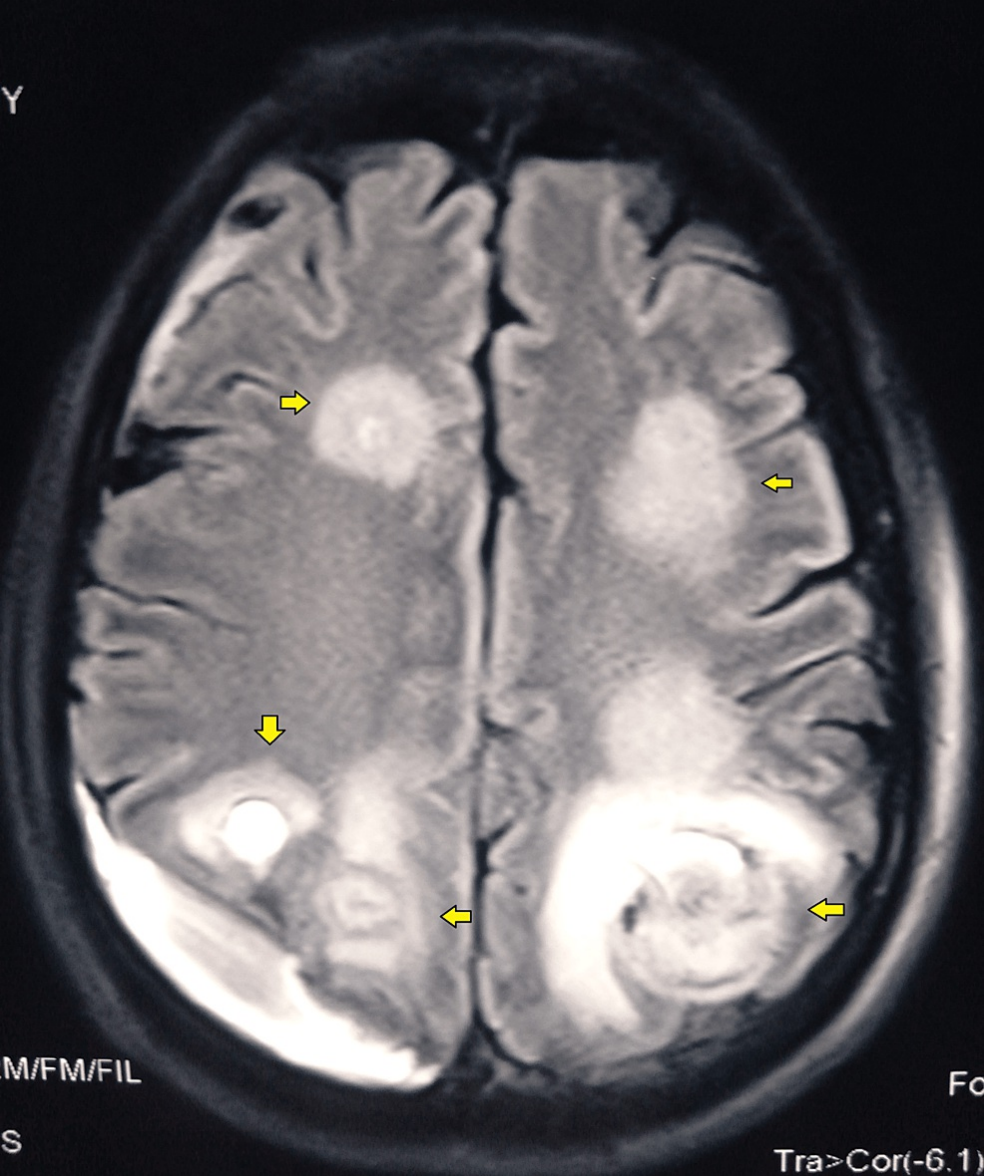
MRI brain ( FLAIR sequence) showing predominately hyperintense lesion involving fronto-parieto-temporo-occipital lobes (yellow arrow) FLAIR- fluid-attenuated inversion recovery

MRI brain with contrast with perfusion imaging revealed hemorrhagic encephalitis involving both the fronto-temporo-parieto-occipital lobes involving the cortical, subcortical, and splenium of the corpus callosum, the posterior limb of the right internal capsule. Subacute subdural hemorrhage at the right temporo-parieto-occipital convexity (Figure 3).

**Figure 4:**
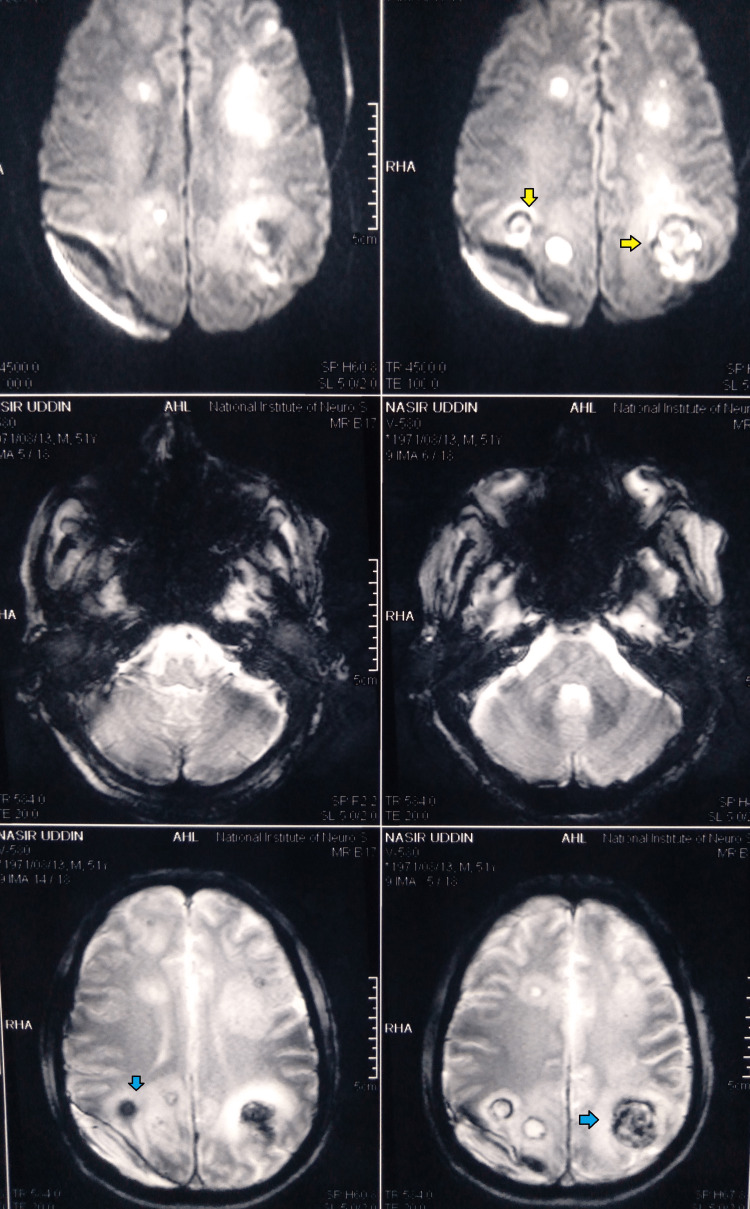
MR perfusion scan shows diffusion restriction in DWI (yellow arrow) and blooming in GRE sequence (blue arrow) DWI- diffusion-weighted imaging; GRE- gradient recalled echo

MR perfusion imaging showed partial diffusion restriction restricted areas in both fronto-temporo-parieto-occipital lobes, and gradient recalled echo (GRE) revealed blooming in the above-mentioned area (Figure 4).

Magnetic resonance angiography (MRA) revealed marginal irregularity and a beaded appearance noted at both middle cerebral arteries with distal paucity. However, ICA, ACA, and PCA were found completely normal. MRV was unremarkable.

As the patient’s condition was critical, he was shifted to the intensive care unit and treated with fresh frozen plasma, red blood cell concentrate, broad-spectrum antibiotics (meropenem and moxifloxacin), and methylprednisolone. Although coagulation abnormality was corrected, however, the patient died on the 17th day of hospitalization probably due to multiorgan failure and brain stem herniation.

## Discussion

Here, we present a case of acute hemorrhagic leucoencephalitis triggered by *Mycoplasma pneumoniae*, the first-ever case reported from Bangladesh. This patient presented with severe pneumonia; subsequently, he developed encephalitis, autoimmune hemolytic anemia, and DIC. Pfausler B et al. [3] reported Mycoplasma pneumoniae-related AHLE where both of the patients presented with a short history of upper respiratory tract infection followed by focal neurological deficits like flaccid quadriparesis with unilateral blindness but Rick M et al. [7] described a similar history of upper airway infection followed by an altered level of consciousness with right-sided hemiparesis. Our patient had a similar clinical presentation without any hemiparesis or blindness. The reason behind that was the lesions in the cerebral cortex spare premotor, motor area, and spinal cord. CNS involvement occurs due to an autoimmune reaction, meaning the development of an autoantibody that reacts with myelin and neural tissue is responsible for the disease [3,7]. Tsiodras S et al. reported that the mean interval of CNS manifestation from respiratory infection is nine days (4-14 days) [8]. CNS presentation occurred on the 10th day in our patient. Our patient presented with extrapulmonary features like hemolysis, thrombocytopenia, elevated liver enzymes, raised bilirubin, and disseminated intravascular coagulation. Common extrapulmonary manifestations are described by Kashyap S and Sarkar M [9]. The diagnosis of *M. pneumoniae* is a bit challenging because of the limitations of laboratory tests like difficulty in collecting the sample from the lower respiratory tract. A special technique is needed for culture growth, although sensitivity is only 30-60% [10]. So serological testing is the common mean of diagnosing mycoplasma infection and a four-fold rise in titer between acute and convalescent sera is still considered a "gold standard" to diagnose acute *M. pneumoniae *respiratory infections. Complement fixation tests, enzyme-linked immunoassay, and antigen detection by PCR, immunofluorescence, or immunoblotting are alternative methods of diagnosing mycoplasma infection [9,11]. As we have some limitations in diagnosing mycoplasma infection, we went for a blood culture along with ELISA for detecting circulating antibodies. Blood culture was found negative but ELISA revealed a four-fold rise in immunoglobulin G (IgG) antibody. To exclude other viral infections, including COVID-19, multiplex PCR from CSF was done and found negative. The core diagnostic feature for mycoplasma-induced AHLE is fibrinoid necrosis in the vessel wall with positive immunohistochemistry findings of brain parenchyma [7]. In our patient, it was not possible to perform a brain biopsy and the autopsy was not done due to a religious perspective. Another strong differential of AHLE is acute demyelinating encephalomyelitis. Sometimes, it is difficult to distinguish AHLE from ADEM. We made ADEM, cerebral venous thrombosis, CNS vasculitis, and hemorrhage due to DIC the imaging differentials of the patient. We excluded ADEM by the nature of the MRI lesions; the perilesional edema of the lesions and associated hemorrhage helped in the exclusion. Brain MRI is highly important and typically reveals confluent white matter lesions with significant edema, petechial hemorrhages, and space-occupying effects. However, there is variability regarding the location of the lesions. Approximately, two-thirds of patients had uni- or bilateral hemispheric involvement, but other distributive patterns have been described. The most useful aspect of cerebral MRI in differentiating AHLE from ADEM is, however, the presence of intraparenchymal hemorrhages [1,6]. Therefore, considering the clinical history, positive laboratory, and radio-imaging (chest X-ray, MRI brain with magnetic resonance venography (MRV), and MRA with contrast) findings diagnosis was made. Treatment of AHLE is immunosuppressive therapy like steroids, cyclophosphamide, rituximab, and plasmapheresis. Even after treatment prognosis is poor. Mortality is around 46.5%, whereas only 14% of patients made a full recovery and 39.5% survived with mild to severe neurological impairment [1]. Although IV methylprednisolone, broad-spectrum antibiotics, and fresh frozen plasma were given for DIC due to severe sepsis, the patient died on the 17th day of his illness. Probably brain stem herniation and sepsis were responsible for the cause of his death. Even after aggressive treatment, mortality is high in AHLE. So early diagnosis and initiation of immunosuppressive therapy are the cornerstones of management.

## Conclusions

*Mycoplasma pneumoniae* should be considered a potential cause of encephalitis in adults, especially those with a preceding history of acute respiratory tract infection. Serological testing is more reliable for the diagnosis of the association between encephalitis and *Mycoplasma pneumoniae.*
